# Sex disparities in the association between rare earth elements exposure and genetic mutation frequencies in lung cancer patients

**DOI:** 10.1038/s41598-024-79580-z

**Published:** 2025-01-16

**Authors:** Mengyuan Liu, Jiali Zhang, Xiaohong Duan, Qiming Zhou, Jing Chen, Siyao Liu, Junyan Su, Li Han, Fan Yang, Niansong Qian

**Affiliations:** 1 Department of Oncology, Senior Department of Respiratory and Critical Care Medicine, The Eighth Medical Center of Chinese PLA General Hospital, No.17 A Heishanhu Road, Haidian District, Beijing, 100853 China; 2 Computer Network Information Center, Chinese Academy of Sciences, Beijing, 100190 China; 3 WillingMed Technology (Beijing) Co., Ltd, Beijing, 100176 China; 4 Beijing ChosenMed Clinical Laboratory Co., Ltd, Beijing, 100176 China; 5 Department of Oncology, The Fifth Medical Center of Chinese PLA General Hospital, Fengtai dong Road, Fengtai District, Beijing, 100853 China

**Keywords:** Rare earth elements, Genetic mutations, Lung cancer, Sex-specific disparities, Environmental sciences, Oncology

## Abstract

The ubiquitous use of rare earth elements (REEs) in modern living environments raised concern about their impact on human health. With the detrimental and beneficial effects of REEs reported by different studies, the genuine role of REEs in the human body remains a mystery. This study explored the association between REEs and genetic mutations in patients with lung adenocarcinoma (LUAD). A cohort of 53 LUAD patients underwent tumor DNA sequencing (1123 cancer-related genes) and plasma REE (lanthanum (La), cerium (Ce), praseodymium (Pr), neodymium (Nd), and yttrium (Y)) quantification. We found divergent relationships between plasma REE levels and mutation load between sexes. Specifically, Ce levels and mutation load were positively correlated in males but negatively correlated in females, while La exposure exhibited opposite associations in the two sexes. This observation was validated using the Bayesian Kernel Machine Regression (BKMR) model. Additionally, plasma REE levels was associated with specific mutation types and variant allele frequencies (VAFs) of particular genes in a sex-dependent manner. Mutational signature analysis revealed sex-specific associations of La with indel signatures. These findings highlight the intricate interplay between plasma REE levels and genetic mutations in LUAD, emphasizing the need for a personalized, sex-oriented approach to understand and treat this disease.

**Supplementary Information:**

The online version contains supplementary material available at 10.1038/s41598-024-79580-z.

## Introduction

The rare earth elements (REEs) consist of 14 lanthanides, as well as scandium and yttrium, which exhibit similar chemical properties to lanthanides. The widespread applications of REEs in industrial, agricultural, and iatrogenic technologies have raised concern about their impact on human health, as reflected by the growing literature on REE-related adverse effects^1^. To our knowledge, no essential biological function of these elements and their compounds has been established. A body of experimental studies have suggested that high level of REE exposure exerts a damaging effect on multiple in vivo or in vitro models^2–6^. However, the exact biological impact of REEs is still an unsolved controversy. The beneficial effects of REEs have been documented in many studies, as the REEs have been supplemented in fertilizers in agriculture practice to improve plant health and used as feed additives in veterinary to boost animal production^7,8^. Thus, the exact function of REEs in organic systems needs to be further elucidated.

Epidemiological studies on the association between REEs and diseases have increased in recent years. The high exposure levels of REEs have been related to the risk of disrupted fetal development^9,10^. For housewives in Shanxi province of China, higher concentrations of hair REEs were associated with hypertension^11^. The application of Gd as a contrast agent in iatrogenic practice can cause severe damage to brain tissue and give rise to nephrogenic systemic fibrosis^12^. However, on the other end, studies on human population also documented the beneficial effects of REEs. Higher levels of serum REEs during early pregnancy were related to a decreased risk of gestational diabetes mellitus^13^. A recent study demonstrated that prenatal exposure to REEs was positively associated with the telomere length of newborn cord blood^14^. Moreover, Ce has been reported to act as an antioxidant to scavenge ROS at physiological pH and as a pro-oxidant to generate ROS under low pH environments in cancer cells^15^. In concordance with this finding, Ce concentration was linked with a lower risk for oral cancer in a recent study^16^. Another study showed a U-shaped relationship between serum La levels and oral cancer risk, suggesting a protective role of La under low concentrations^17^.

The etiology of cancer is highly complex, and the genetic mutations that lead to activation oncogenes or suppression of cancer suppressor genes have long been considered a major contributor to carcinogenesis. In recent years, progressions in high-throughput DNA sequencing techniques have dramatically broadened our knowledge about cancer occurrence and development mechanisms from the molecular perspectives. However, the link between REE exposure and genomic variation is still a knowledge gap in the current field of cancer research.

Lung cancer has been regarded as a distinct disease in women related to that in men. Besides the distinct environmental exposures that are imposed as different risk factors for lung cancer, sexual hormones such as estrogen, testosterone, and androgen could also related to lung cancer development in different sexes^18,19^. The expression of the estrogen receptor was reported to be elevated in many lung cancers, and the use of hormonal therapy was associated with a decreased risk for lung cancer in non-smoking women^20^. REEs have been known to promote growth in animals through their influence on hormone secretion, especially the hypothalamic-pituitary-thyroid axis^21,22^, which is tightly related to the hypothalamic-pituitary-gonadal axis^23^. A high dose of Ce exposure in mice could directly result in reduced serum concentration of testosterone^24^. Although the mechanism of how REEs influence the sexual hormone system remains unclear, studies have identified different accumulation and health impacts of REEs between the two sexes^22,25,26^. Based on the best of our knowledge, no previous study has reported the implication of REE in genetic mutations among lung cancer patients or even the sex-based disparities in this carcinogenic process.

La, Ce, and Y constitute roughly 63% of the overall exposure of the 16 REEs^27^, and La, Ce, Pr, Nd, and Y are the most abundant REEs in the street dust of central China^28^. In this study, we aspired to investigate the association between these five lanthanides and genetic mutation in cancer patients, shedding light on the impact of REE exposure on the genome stability involved in cancer development. A large next-generation sequencing (NGS) panel (consisting of 1123 cancer-related genes) was utilized to detect gene mutations. Meanwhile, we adopted the plasma REE concentration as a proxy for whole-body REE exposure level. The plasma concentrations of La, Ce, Pr, Nd, and Y were assessed using inductively coupled plasma mass spectrometry (ICP-MS). Applying the Bayesian kernel machine regression (BKMR) model, we found a negative association between the joint exposure level of these REEs and the frequencies of genetic variations.

## Materials and methods

### Participant selection and sample collection

We enrolled a group of patients diagnosed with lung adenocarcinoma (LUAD). Clinical information, DNA sequencing data, and plasma concentration were obtained and investigated retrospectively. Considering the mutation happens and accumulates along with age^29,30^, it is necessary to regard age as a confounder in elucidating the association between genetic variation and environmental exposure. Thus, we restricted the age of included patients to 60–80 years. Only the patients whose tumor tissue was tested by the ChosenMed^®^ 1123-gene (ChosenMed Technology, Beijing, Co., Ltd.) test were included. The samples were obtained through surgical procedures or biopsy prior to any other medical treatments. We excluded the patients who were first diagnosed as cancer before 60 years old or possessed any germline cancer susceptible genetic variation or a cancer history in their direct relatives. Sex was not a criterion in the selection of eligible participants.

This study was approved by the ethics committee of China Human Genetic Resources Management Office, Ministry of Science and Technology of the People’s Republic of China, and the reference number for approval was 22021SLCJ1864. We have received the informed consent from all participants before the investigation. All methods were performed in accordance with the relevant guidelines and regulations.

### Laboratory assessment of the REEs

The detailed methods for laboratory assessment have been described in our previous work^31^. In brief, ten milliliters of peripheral blood from each patient was collected in the anticoagulant collection vessel. The blood was then centrifuged, and the resulting plasma was separated and stored in a -80 °C refrigerator. The plasma was taken to 4 °C to thaw prior to the analysis, and 0.1 milliliter was pipetted to a centrifugal tube. For quality control, 0.1 milliliter mixture containing indium and rhenium was supplemented as inner reference substances. The plasma next underwent digestion by nitric acid (UP grade), followed by thorough mixing before being analyzed by inductively coupled plasma mass spectrometry (ICP‒MS, ELAN DRC II, Perkin-Elmer Sciex, Wellesley, MA, USA). This quantitative assessment of plasma REEs was conducted by Peking University Biomedical Analysis Center, with a standard operational procedure approved by China Metrology Accreditation (CMA). The experimental detect limits were 0.0006 ng/mL, 0.0004 ng/mL, 0.0001 ng/mL, 0.0003 ng/mL, and 0.001 ng/mL for La, Ce, Pr, Nd, and Y, respectively. The detection rate of all the assessed REEs in this study cohort reached 100%.

### DNA sequencing by NGS and mutation calling process

The methods involved in DNA sequencing and mutational calling have also been provided in our previous work^31^. In brief, we isolated the tumor DNA of each participant from five formalin-fixed paraffin-embedded (FFPE) sections following the producer’s guidelines for the FFPE tissue DNA extraction kit (Concert, Cat: RC1004). The genomic DNA (gDNA) of each patient was obtained from peripheral blood and taken as a background. Quality and quantity assessment of the extracted DNA was performed using Qubit (Thermo, Cat: Q33231), followed by being fragmented by ultrasound (Covaris M220). The resulting DNA fragments were then processed to generate libraries for gene NGS, according to the manual of KAPA HyperPrep Kits (KAPA, Cat: KK8504). Gene sequencing was performed using a panel containing probes for 1123 genes (ChosenMed^®^ 1123 panel, 2.2 Mb) with the MGI-2000 sequencer (BGI)^32^. We produced 8G data for tumor somatic DNA, maintaining a minimum depth of 2000x, and 2G data for gDNA.

After excluding low-quality reads, eliminating primers, and trimming adaptors, we employed the Burrows-Wheeler Aligner (BWA, RRID: SCR_010910, version 0.7.11) to align the sequence to the reference genome (hg19) and generate BAM files^33^. Throughout all procedures in this study, investigators were maintained inaccessible to patient information to ensure a blind methodology.

The software VarDict (RRID: SCR_023658), MuTect2 (RRID: SCR_000559), and VarScan (RRID: SCR_006849) in the GATK module were employed to identify somatic variation. The variation was kept for further analyses when it received more than or equal to one “PASS” derived by the above software. Discerning SNVs and indels relied on a series of filtering criteria, which has been mentioned in our previous work^31^.

### Statistical analysis

The extreme values were substituted by the winsorization method^31,34^. The concentration of each element was examined for normal distribution by Shapiro-Wilk test, and the concentration of La and Y were not normally distributed. Thus, the comparisons of element concentrations were conducted using Wilcoxon test, while the correlations between variables were examined using Spearman correlation.

Bayesian kernel machine regression (BKMR) was employed to examine the combined impact of the REEs. This model was created to explore the integrated effect of several influencers that the subjects commonly encounter synchronously^35^, which cannot be revealed by traditional statistical methods. The BKMR modelling framework is Y_i_=h(La, Ce, Pr, Nd, and Y)+ ß^T^Z_i_ + e_i_. In this formula, h(⋅) is referred as the exposure-response function; Zi is a pack of covariates that should be adjusted as confounders; ß presents the corresponding vector of the covariates. Firstly, we harnessed this model to assess the overall effect of the REEs as a co-exposure mixture by examining the variation in the parameter ‘h’ when all exposures are at a specific percentile versus when they are at the median (50th percentile). Second, we can summarize the individual effect of each exposure to response by contrasting the value of ‘h’ when the exposure is at the 75th percentile to when the exposure is at its 25th percentile, while maintaining the other exposures at a designated percentile. Additionally, we examined each element’s univariate relationship with the response, when keeping the others at their 50th percentile. Before being input into the model, the element concentrations were normalized using z scores after logarithmic transformation.

The mutational signatures of SNVs and indels were deconvoluted with a Python package “SigProfilerExtractor (version 3.8)”^36^. This framework allows De Novo extraction of mutational signatures and the decomposition into the existing COSMIC signature. This extraction process employs nonnegative matrix factorization (NMF) to decompose a mutation matrix into two low-rank matrices: one for signatures and one for their activities, facilitating biological interpretation of the non-negative factors.

We use R (v 4.2.2) to analyze and visualize the data. A dual-tailed p-value below 0.05 was recognized as an indication of statistical significance. The p-values were adjusted for multiple comparisons using the false discovery rate (FDR) method and are presented as p-adj.

## Results

### Comparison of REE exposure and mutation load between two sexes

**Fig. 1:**
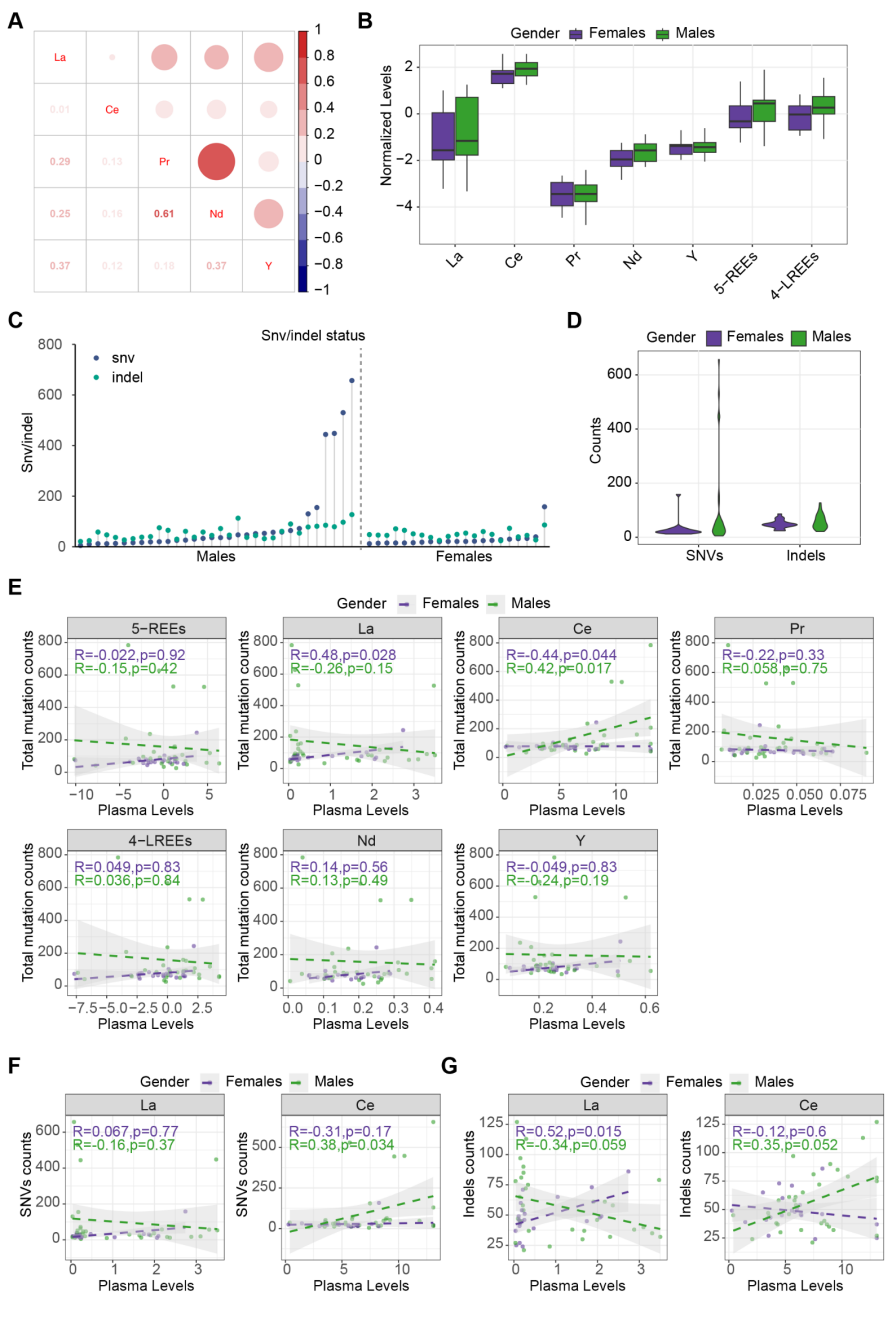
The interrelationships between REE concentrations, sexes, and gene mutations. (**A**) Correlations between plasma concentrations of five REEs. The numbers show the value of coefficient r of the correlation examination. (**B**) Comparisons of the assessed REEs between two sexes. (**C**) Single nucleotide variations (SNVs) and insertions/deletions (indels) count of each participant, sorted by SNV number in two sexes. (**D**) Comparison of SNV and indel number between the two sexes. (**E**) Correlations of the five individual REEs, the sum of scaled REE concentrations of the five REEs (5-REEs), and the four light REEs (4-LREEs) with the total mutation numbers, comparing between the two sexes. (**F**) Correlations of La and Ce with SNV counts, comparing between the two sexes. (**G**) Correlations of La and Ce with indel counts, comparing between the two sexes.

With the inclusion criteria listed in the methods section, 53 lung adenocarcinoma (LUAD) patients were enrolled for tumor DNA sequencing and plasma REE concentration quantification. Information on smoking status and cancer stage could not be obtained from the participants, which may act as potential confounders. The five elements showed weak or moderate correlation with each other (Fig. 1A). The ages of the male and female groups were similar (Table 1). After removing outlier values, we conducted a Wilcoxon test to compare the levels of the examined REEs (La, Ce, Pr, Nd, and Y) between the two sexes. We observed that Nd was significantly higher in male than female patients before multiple testing correction (male vs. female 0.21 (0.13–0.28) vs. 0.14 (0.11–0.21) ng/mL, *p* = 0.029, p-adj = 0.145), and the level of Ce was also elevated in males, although statistically nonsignificant (male vs. female 6.96 (5.13–9.04) vs. 5.57 (3.68–6.41) ng/mL, *p* = 0.088, p-adj = 0.22) (Table 1; Fig. 1B). The combined levels of the five REE elements and the four light REE elements (La, Ce, Pr, and Nd) were also calculated, and a significantly higher level of combined four light REEs was noted in male than in female patients (*p* = 0.046, Table 1; Fig. 1B).

For genetic analysis, we performed DNA sequencing on tumor DNA and matched gDNA (from peripheral blood) with an NGS panel consisting of 1123 genes associated with cancer. Each sample had an average coverage depth exceeding 2000x. After variant calling processes, we confirmed 3,796 SNVs and 2,851 indels in the tumor somatic DNA. The male patients exhibited more SNVs (*p* = 0.042, Table 1; Fig. 1C and D). This disparity could be explained by the potentially higher proportion of smokers among male patients since smoking is a known factor that leads to higher mutation rates in lung tumors^37^.

**Table 1:** Comparison of age and REE levels of male and female patients.

	Male (*n* = 32)	Female (*n* = 21)	*p*	*p*-adjust
Age (years, Median (P25–P75))	66 (64-70.25)	68 (65–72)	0.412	
Metal exposures (Median (P25–P75), ng/mL)				
La	0.31 (0.17–2.04)	0.21 (0.14–1.05)	0.144	0.240
Ce	6.96 (5.13–9.04)	5.57 (3.68–6.41)	0.088	0.220
Pr	0.03 (0.02–0.05)	0.03 (0.02–0.05)	0.698	0.873
Nd	0.21 (0.13–0.28)	0.14 (0.11–0.21)	**0.029**	0.145
Y	0.24 (0.19–0.29)	0.25 (0.18–0.27)	0.878	0.878
Combined exposure after normalization				
Five REEs	1.03(-1.21-1.45)	-1.2(-2-0.74)	0.073	
Four light REEs	0.51(-0.29-1.89)	-0.34(-2.28-0.73)	0.046	
*Mutation rates (Median (P25–P75))*				
SNVs	36.0 (18.5–59.5)	21 (16–28)	**0.042**	
Indels	50.50 (37.00-75.75)	46 (41–54)	0.507	

### The sex disparities of the relationships between REE concentration and genetic mutations

Specific REEs exhibited moderate correlations, with the most robust relationship between Pr and Nd (*r* = 0.61 p-adj < 0.001), followed by Nd and Y (*r* = 0.47, p-adj = 0.002) and La and Y (*r* = 0.40, p-adj = 0.011, Fig. 1A), suggesting similar assimilation and metabolism routines of these REEs. Within the constricted age range of the participants, both the five REEs and the mutation number showed no significant association with age (Fig S1A and B). Next, we investigated the correlation between REE concentration and total mutation numbers. The results showed a divergent relationship between Ce and mutation load between male and female patients (Fig. 1E). The mutation loads were increased with Ce exposure in males (*r* = 0.42, *p* = 0.017, p-adj = 0.353) while showing a negative correlation in females (*r*=-0.44, *p* = 0.044, p-adj = 0.353). A similar phenomenon was also found in La, with a significant positive correlation between La exposure and mutation frequencies in females (*r* = 0.48, *p* = 0.028, p-adj = 0.353) and a suggestive inverse relationship in males (*r*=-0.26, *p* = 0.15, p-adj = 0.488). We next examined the correlation between REE exposures and the number of SNVs and Indels and found similar trends of divergencies between the two sexes, albeit some of them were not statistically significant (Fig. 1F and G).

###  Integrated and respective influences of REEs on DNA mutations using the BKMR model and their sexual disparities

We employed the BKMR model^35^ to investigate the integrated influences of the assessed REEs and dissect the individual effects of each element while accounting for the impact of other REEs. Since we have observed different correlations of REEs with mutation burden between sexes, we conducted the BKMR analysis separately for different sexes. Meanwhile, we investigated the respective influence of REEs on SNVs and indels.

**Fig. 2:**
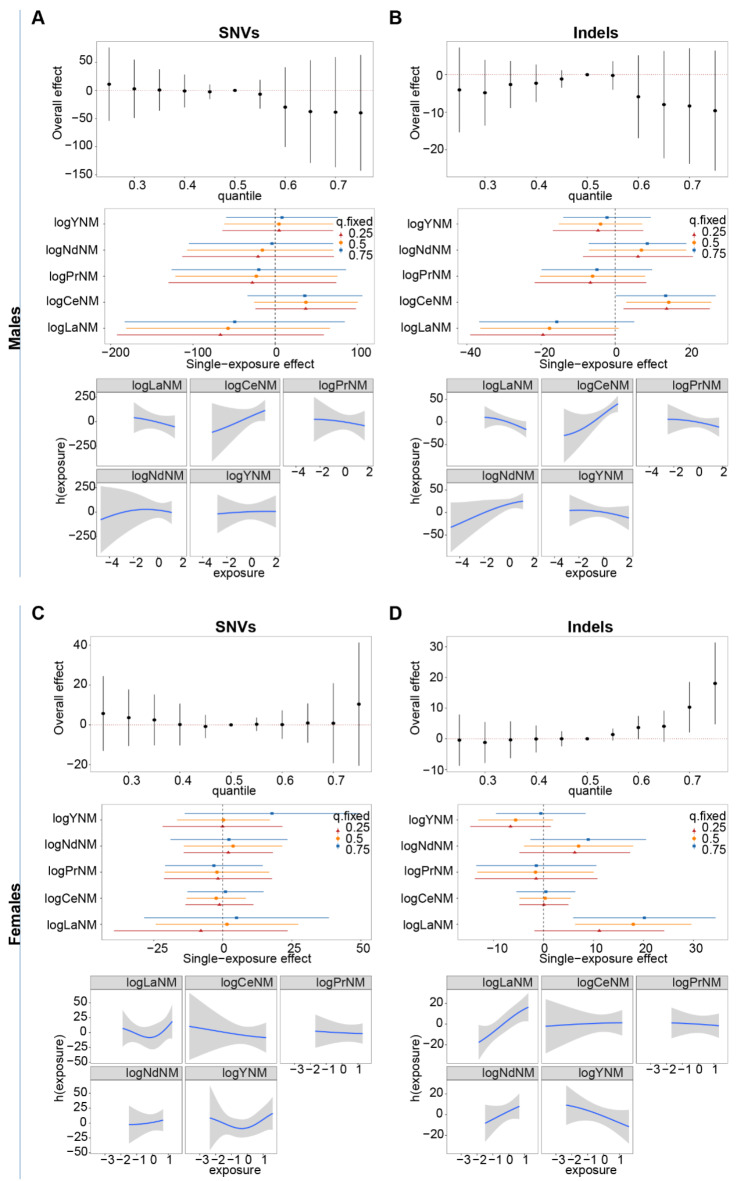
Association between integrated five REE exposures and the number of gene mutations analyzed using the Bayesian kernel machine regression (BKMR) (SNV number in males (**A**) and females (**C**), and indel number in males (**B**) and females (**D**)). For each figure, the **upper** image shows the integrated influences of the assessed REEs on the total incidence of gene mutations when the entire concentrations changed across the 25% percentile to the 75% percentile as compared to the 50% percentile; the **middle** image illustrates the respective impact of each element on gene mutation number while holding the other four REEs at their 25%, 50%, or 75%^th^ percentile; the **lower** image exhibits the continuous influence of individual REE on the gene mutation number when the other four REEs were maintained at their median levels. The element concentrations used in this analysis were normalized using z-scores after log transformation. These figures illustrate estimations and 95% confidential intervals while controlling for age as covariates.

We derived three parts of results from BKMR analysis. First, the integrated influences were evaluated when the overall concentrations of the five REEs changed across the first quantile (25th ) through the third quantile (75th ) as compared to the median (50th ) level, with age being adjusted as the confounder (Fig. 2. **upper images in all figures**). We found that the number of indels increased with the combined exposure level in female patients, and the association was significant when the overall concentration was above the 60th, 70th, and 75th percentile (Fig. 2D **upper**). In the second part, the model can decipher the respective impact of each element on mutation number by holding the other elements at their 25th, 50th, and 75th percentile (Fig. 2. **middle images in all figures**). We observed a significant positive association between Ce (at all three percentiles) and indel mutation in males (Fig. 2B **middle**), and between La (at 25th and 50th percentile) and indel mutation in females (Fig. 2D **middle**). There was no association between the other three REEs and mutation numbers. In the third part, when the other four REEs were maintained at median levels, the model gives the exposure-response function of each REE with the mutation numbers (Fig. 2. **lower images in all figures**). The results showed that Ce was positively correlated with SNV and indel numbers in males (Fig. 2A and B **middle**), and La was associated with indel numbers in females (Fig. 2D **middle**).

### Associations between REE concentration and specific genetic variation types in two sexes

**Fig. 3:**
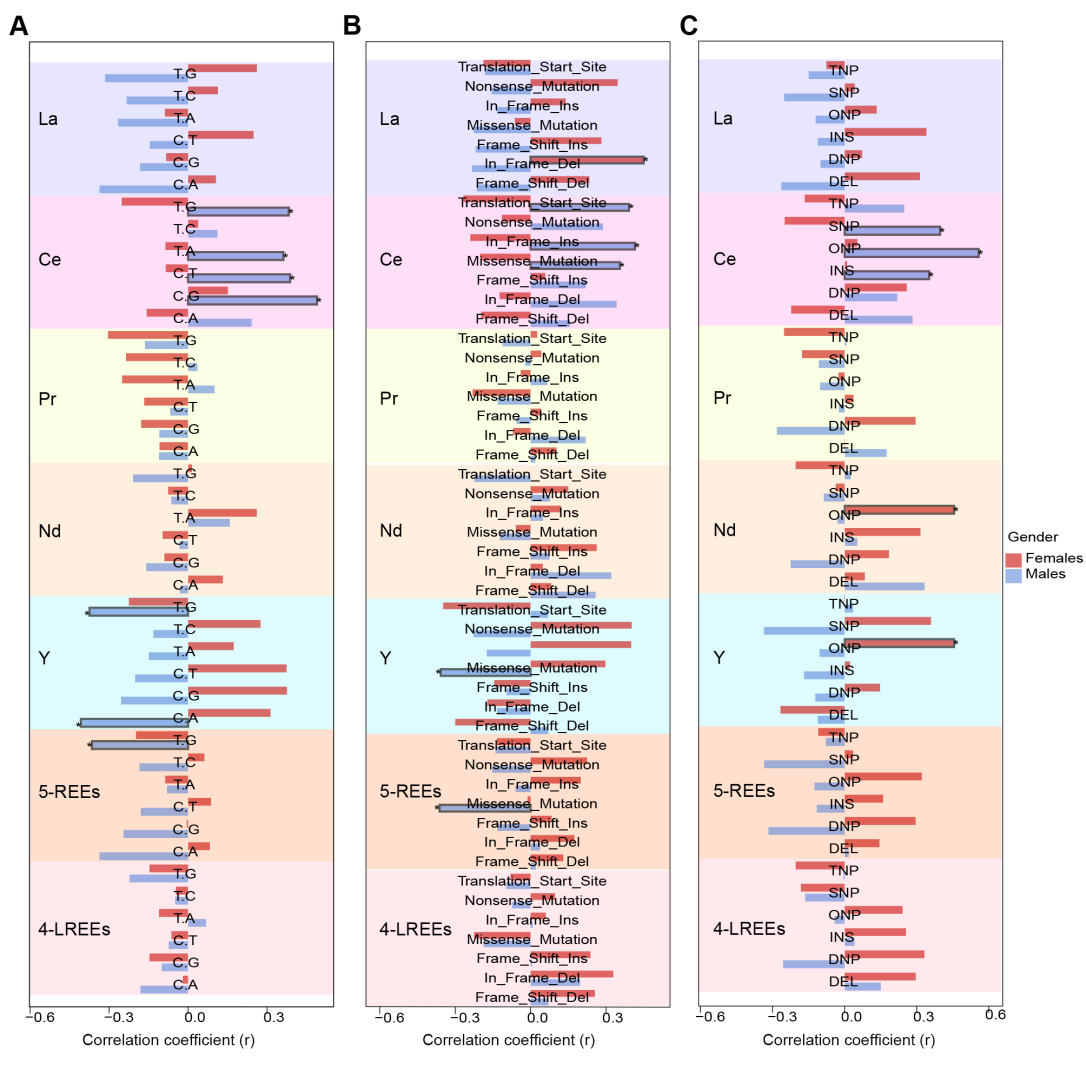
Correlations between REE concentration and specific genetic variation types, comparing between two sexes. **(A)** Correlations between REE and six SNV classes, including T > G, T > C, T > A, C > T, C > G, and C > A. **(B)** Correlations between REE and seven variant types, including translation starting sites, nonsense mutations, in-frame insertions, missense mutations, frameshift insertions, in-frame deletions, and frameshift deletions. **(C)** Correlations between REE and six variant classifications, including TNP (Triple nucleotide polymorphism), SNP (Single nucleotide polymorphism), ONP (Oligo-nucleotide polymorphism), INS (Insertion), DNP (Double nucleotide polymorphism), and DEL (Deletion). Columns with dark frames indicate statistical significance (*p* < 0.05).

Based on the observations of the relationships between REE exposure and mutational load, we subsequently sought to ascertain whether specific types of variations might be particularly influenced by exposure to the detected REEs. We categorized these mutations in three distinct manners, namely “SNV class”, “Variant Type”, and “Variant classification”. In male patients, we found that the Ce level was significantly associated with rates of C > G (*r* = 0.490, *p* = 0.004, p-adj = 0.724), C > T (*r* = 0.388, *p* = 0.028, p-adj = 0.724), T > A (*p* = 0.362, *p* = 0.042, p-adj = 0.724), and T > G (*r* = 0.382, *p* = 0.031, p-adj = 0.724) mutation (Fig. 3A). Meanwhile, the number of missense mutations (*r* = 0.355, *p* = 0.046, p-adj = 0.724), in-frame insertions (*r* = 0.415, *p* = 0.018, p-adj = 0.724), and mutations in translational starting sites (*r* = 0.392, *p* = 0.027, p-adj = 0.724) also increased with Ce exposure (Fig. 3B). Consistently, the frequencies of insertion (INS, the addition of nucleotides) (*r* = 0.351, *p* = 0.049, p-adj = 0.724), single nucleotide polymorphism (SNP, a substitution in one nucleotide) (*r* = 0.397, *p* = 0.024, p-adj = 0.724), and oligo-nucleotide polymorphism (ONP, a substitution in more than three consecutive nucleotides) (*r* = 0.558, *p* < 0.001, p-adj = 0.331) were higher in patients with elevated Ce plasma concentration (Fig. 3C). Moreover, the concentration of Y was negatively correlated with the number of C > A (*r*=-0.407, *p* = 0.021, p-adj = 0.724), T > G (*r*=-0.374, *p* = 0.035, p-adj = 0.724), and missense mutations (*r*=-0.358, *p* = 0.044, p-adj = 0.724) in males (Fig. 3A and B). However, in female patients, Ce and Y exhibited opposite relationships with these mutation classifications, albeit not statistically significant. A significant correlation was observed in females between La exposure and in-frame deletion (*r* = 0.450, *p* = 0.041, p-adj = 0.724), where the males showed a divergent trend (*r*=-0.233, *p* = 0.200, p-adj = 0.792) (Fig. 3B).

### Association between REE exposure and frequencies of genetic variation and mutational signatures

**Fig. 4:**
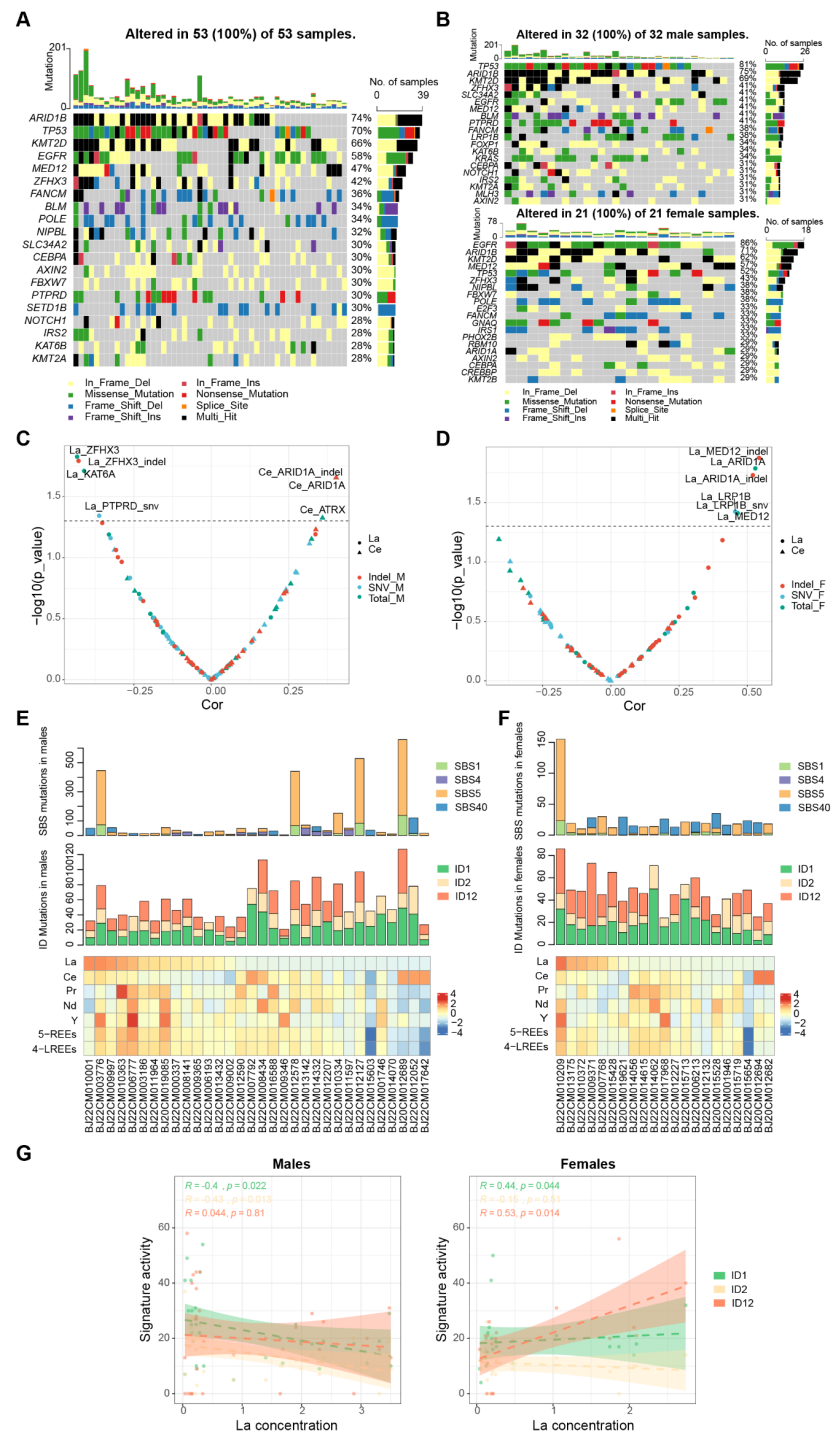
(**A**) Mutational landscape showing the mutation types and rates of most frequently mutated genes in the overall cohort. (**B**) Mutational landscape showing the mutation types and rates of most frequently mutated genes in male patients (upper image) and female patients (lower image). Correlation of the variant allele frequencies (VAFs) of highly mutated genes and La and Ce in male patients (**C**) and female patients (**D**). The x-axis and y-axis represent the correlation coefficient (rho) and log-transformed P value. The absolute contributions of the SBS and ID mutational signatures for the mutation spectrum of each patient are displayed in male patients (**E**) and female patients (**F**), along with the corresponding levels of REEs (sorted in descending order of La). (**G**) The correlation of the three ID signatures (ID1, ID2, and ID12) and La concentration in male (left) and female (right) patients.

The most frequently mutated genes in our cohort were identified, with ARID1B, TP53, KMT2D, EGFR, MED12, ZHX3, FANCM, and BLM ranking at the top of the list (Fig. 4A). However, specific genes show distinct mutation rates between the two sexes. EGFR mutations were more frequently present in female patients (M vs. F, 41% vs. 86%), while the TP53 mutation was enriched in male patients (M vs. F, 81% vs. 52%) (Fig. 4B), which echoes the data in previous studies^38,39^. Next, we examined the association between gene variation allele frequencies (VAFs) and REE exposure levels. For males, Ce was positively associated with VAFs of ARID1A (*r* = 0.403, *p* = 0.022, p-adj = 0.597), ATRX (*r* = 0.359, *p* = 0.047, p-adj = 0.725) and ARID1A indels (*r* = 0.403, *p* = 0.022, p-adj = 0.597), and La was negatively correlated with VAFs of ZFHX3 (*r*=-0.433, *p* = 0.015, p-adj = 0.597), ZFHX3 indels (*r*=-0.429, *p* = 0.016, p-adj = 0.597) KAT6A (*r*=-0.411, *p* = 0.019, p-adj = 0.597), and PTPRD SNVs (*r*=-0.362, *p* = 0.045, p-adj = 0.725) (Fig. 4C). For females, the La concentration was positively associated with VAFs of MED12 (*r* = 0.465, *p* = 0.039, p-adj = 0.725), MED12 indels (*r* = 0.543, *p* = 0.013, p-adj = 0.597), ARID1A (*r* = 0.530, *p* = 0.016, p-adj = 0.597), ARID1A indels (*r* = 0.521, *p* = 0.019, p-adj = 0.597), LRP1B (*r* = 0.456, *p* = 0.038, p-adj = 0.725), and LRP1B SNVs (*r* = 0.456, *p* = 0.038, p-adj = 0.725) (Fig. 4D).

To investigate the potential mutational processes that might related to REE exposure, we deconvoluted the mutational signatures. For single base substitution (SBS), four established COSMIC SBS signatures, namely SBS1, SBS4, SBS5, and SBS40, were best fitted for the entire mutational spectrum. For indels (ID), three COSMIC ID signatures, ID1, ID2, and ID12, were extracted. The contribution of these signatures in each participant was illustrated (Fig. 4E and F), and their correlations with REE levels were examined. Interestingly, the concentration of La was negatively associated with ID1 (*r*=-0.4, *p* = 0.022, p-adj = 0.667) and ID2 (*r*=-0.43, *p* = 0.013, p-adj = 0.653) in males, whereas it positively correlated with ID1 (*r* = 0.44, *p* = 0.044, p-adj = 0.838) and ID12 (*r* = 0.53, *p* = 0.014, p-adj = 0.653) in females (Fig. 4E, F and G).

## Discussion

This study investigated the associations between the total number of SNVs and indels in cancer-related genes and five REE (La, Ce, Pr, Nd, and Y) concentrations in lung cancer patients and their sex-based disparities. Interestingly, we found apparent differences in two elements (La and Ce) in their associations with genetic mutation between the two sexes. La exhibited a positive correlation with genetic mutations in female patients but a negative correlation in male patients. These associations were more significant when analyzing the indel mutation separately or the individual established ID mutational signatures. On the other hand, compared to La, Ce showed opposite relationships with genetic mutations in the two sexes. The mutation number increased with Ce in males but decreased in females. These two elements were also associated with the VAFs of specific genes. This study represents the pioneering investigation into the relationship between REEs and genetic mutations among cancer patients.

The current literature lacks information about the standard safe REE concentration in the human body. Only one previous study has reported REE concentration in plasma, although many studies explored the REE concentration in peripheral blood^9,40,41^. Compared to this previous study presented plasma REE concentration in a healthy population, our results showed much higher median concentrations of La (0.36 ng/mL vs. 0.051 ng/mL), Ce (5.87 ng/mL vs. 0.1229 ng/mL), Pr (0.034 ng/mL vs. 0.0101 ng/mL), and Nd (0.19 ng/mL vs. 0.016 ng/mL), whereas much lower median concentration of Y (0.25 ng/mL vs. 0.439 ng/mL)^42^. These differences may be due to the cancerous status of the participants in this study. However, comparing results between different studies might not be plausible because different technologies employed across studies for blood collection and element assessment could introduce bias to the values.

In addition, the results showed a higher plasma concentration of La, Ce, and Nd in males than in females. A similar trend has been observed in previous studies, which reported higher concentrations of REEs in the hair of males than that of females^25,43^. Our results also showed disparate roles of La and Ce in their association with mutation numbers between the two sexes. These results indicated a potential difference in REE metabolic processes and subsequent functions between the two sexes. Blood biochemical indices have suggested a different sensitivity to REE exposure between men and women^44^. Additionally, the different implications of REE exposure in hormone secretion^45^ might also be the underlying mechanism for the observed disparities between the two sexes^46^. However, the precise metabolic pathway and the ensuing roles of REEs in different sexes remain largely elusive, necessitating further investigation.

The REE is a misnomer since REEs are not rare in the earth’s crust^47^. China is the dominant provider of the world’s REEs, followed by Australia and the USA^47^. However, according to a recent epidemiological study in China, the general intake of REEs through dietary for the Chinese population was low and posed a hazard index far below 1^27^. Some studies also investigated the environmental exposure of REEs, which indicated null significant health effects because of their low exposure level^48,49^. Even for those who lived near mining or industrial sites, the estimated daily intake of REEs was significantly lower than the reference threshold (70 µg kg^− 1^d^− 1^), and the health hazards for human beings were negligible^50,51^. In central China, the ambient exposure of REEs presented no augment in cancer or non-cancer risk^28^. Additionally, previous studies have suggested a biphasic relationship between REE exposure and its induced effect, showing beneficial results at low concentrations but toxic effects at high concentrations^52^. Although the concentrations of rare earth elements (REEs) found in this study were higher than those reported in previous research, they may not have reached levels known to induce toxicity. Our findings indicate a negative correlation between the combined levels of the five REEs and the overall number of mutations. This inverse relationship could be attributed to the relatively low exposure levels experienced by the participants in our study, suggesting that at such low concentrations, REEs might exert a protective influence on DNA stability.

Many studies published decades ago have documented the interactions between lanthanide ions and DNA, with lanthanide ions being recognized as catalysts for hydrolysis of phosphate ester bonds of DNA^53^. Recent studies have reported the influence of REE exposure on nucleotide synthesis and epigenetics. A study implied the involvement of REEs in purine nucleotide synthesis, which was downregulated under REE exposure in *Saccharomyces cerevisiae*^54^. For people living near E-Waste recycling sites in southeast China, blood Ce concentration was associated with decreased global DNA methylation level^55^. The genetic and epigenetic activities of REEs might influence the stability of the DNA sequence, and further studies are warranted to elucidate the underlying mechanism of REE’s impact on the DNA mutation found in the current study.

This study provided novel findings about the association between REE exposure and genetic mutations, using a large NGS panel that detects variations in 1123 cancer-related genes. This is the first study to unveil the potential interaction between REEs and genomic stability. Furthermore, we pioneeringly found sexual disparities in the relationships between specific REE and genetic mutation frequencies. However, the shortcomings of this study should also be noted. First, a primary limitation of the study is the use of REE concentrations in plasma as a proxy for exposure, rather than in bone or liver, which would provide a more accurate indication of long-term exposure. This is due to the sample accessibility that we were constrained for using blood samples for the study. Second, smoking status could be a potential confounder in studying the relationship between environmental exposure and genetic mutations. However, we were unable to obtain smoking status information from the participants, and thus no validation of the ideas regarding smoking status can be verified within the current study. Third, ethical constraints prevented us from including healthy individuals as controls, hindering our ability to compare REE concentrations directly between cancer patients and the healthy population within a single cohort without batch effects. Fourth, the DNA variation data were collected from the NGS panel rather than whole-genome sequencing. Therefore, we obtained no information on copy number variations, which are essential to cancer development and progression.

## Conclusion

Our study explored the intricate relationship between rare earth elements (REEs), genetic mutations, and sex-specific differences in lung adenocarcinoma (LUAD). The results indicated contrasting associations between REE exposure and mutation load across sexes, validated by the Bayesian Kernel Machine Regression (BKMR) model. Additionally, REE exposure showed distinct patterns in their relations to specific mutation types, variant allele frequencies (VAFs) of specific genes, and particular mutational signatures. Our findings underscore the complexity of REE-related effects in LUAD and advocate for personalized strategies to address this multifaceted disease. However, this study is rather an exploratory pilot study which suffers from multiple limitations and highlight the need for further investigations specifically planned and designed to answer the questions raised by our findings.

## Electronic supplementary material

Below is the link to the electronic supplementary material.

Supplementary Material 1

## Data Availability

The datasets used and/or analysed during the current study are available from the corresponding author on reasonable request.
