# Supplementary material for: Sex disparities in the association between rare earth elements exposure and genetic mutation frequencies in lung cancer patients
Source: Sci Rep. 2025 Jan 16;15:2185. doi: 10.1038/s41598-024-79580-z (PMC11739476; doi:10.1038/s41598-024-79580-z)
Supplement: Supplementary file 1 — Supplementary Material 1 [file 41598_2024_79580_MOESM1_ESM.pdf]

Supplemental figures

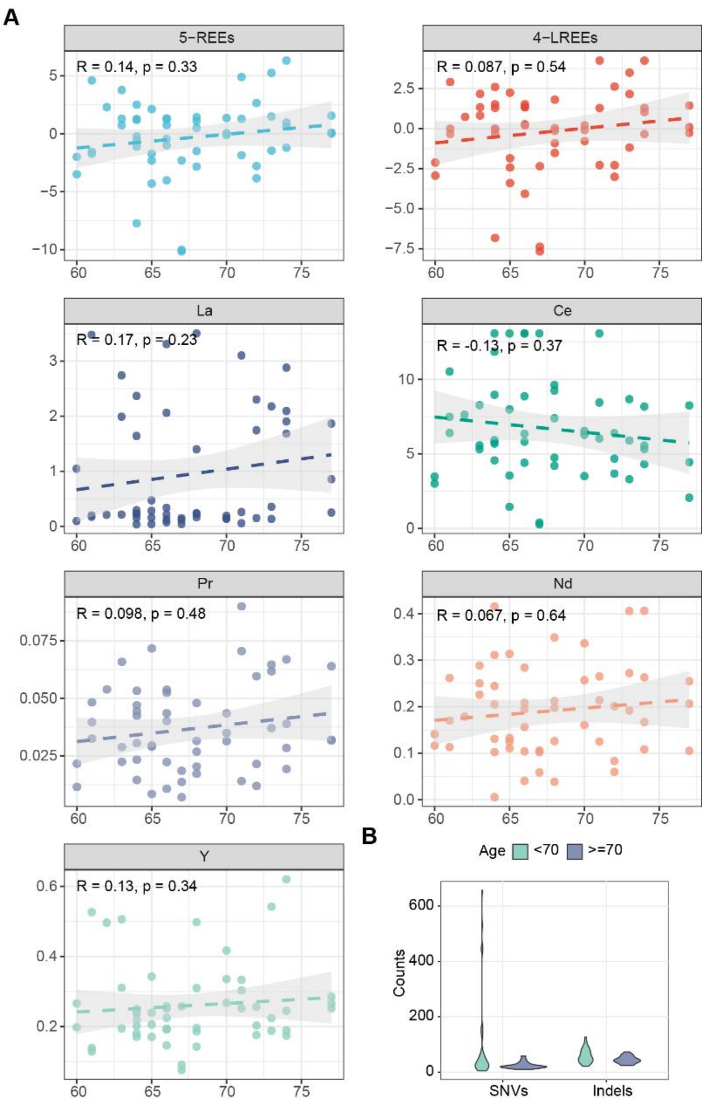

Figure S1. Correlations between age and the five individual REEs, the sum of scaled REE concentrations of the five REEs (5-REEs), and the four light REEs (4-LREEs) **(A)**. Comparison of SNV and indel number between younger (60-70 yrs) and elder (71-80 yrs) patients **(B)**.
